# Endocrinology and Metabolic Diseases in Human Health

**DOI:** 10.3390/nu17071175

**Published:** 2025-03-28

**Authors:** Ilaria Demori, Elena Grasselli

**Affiliations:** 1 Department of Pharmacy (DIFAR), University of Genova, Viale Benedetto XV 3, 16132 Genova, Italy; 2 Department of Earth, Environmental and Life Sciences (DISTAV), University of Genova, Corso Europa 26, 16132 Genova, Italy; elena.grasselli@unige.it

In 2022, we served as guest editors of the Nutrients Special Issue entitled “Endocrinology and Metabolic Diseases in Human Health”. Our contribution focused on the role of stress response in pathogenesis and therapeutic outcomes of hepatic steatosis [1]. At that time, we used the acronym MAFLD (Metabolic dysfunction-Associated Fatty liver Disease) to replace NAFLD (Non-Alcoholic Fatty Liver Disease) [2]. A few years later, a multi-society Delphi consensus statement defined the use of the new term MASLD (Metabolic dysfunction-Associated Steatotic Liver Disease) [3]. Far from being trivial, this evolution in nomenclature reflects a deeper understanding of the pathogenic mechanisms, diverse phenotypic manifestations, and the metabolic comorbidities associated with the disease. As pointed out by Marchesini et al. [4], the new terminology is also intended to raise awareness of the disease and aid in defining therapeutic endpoints.

While the approval of pharmacological agents specifically for MASLD treatment is still pending, novel anti-hyperglycemic drugs show promising potential [5]. Nevertheless, lifestyle interventions targeting energy homeostasis and insulin resistance (IR) continue to be cornerstone strategies for both the prevention and treatment of MASLD and other metabolic disorders.

The bidirectional relationship between nutrition and endocrinology underscores the impact of diet composition and eating habits on human health. In line with this, the retrospective cohort study conducted by Nitta et al. in Japan [6] demonstrated that a simple program of dietary education, led by dieticians, resulted in improved glycemic control after a 5-year period in type 2 diabetes primary-care patients instructed to eat vegetables before carbohydrates. A systematic review of 11 studies [7] confirmed that the consumption of vegetables (and/or proteins) before carbohydrates contributes, at least acutely, to the reduction of glycemic and insulinemic fluctuations. This further emphasizes the evidence that even small behavioral changes in an individual’s lifestyle can have a significant impact on metabolic health. Additionally, increasing dietary fiber intake has been linked to improvements in neurodegenerative symptoms associated with obesity and diabetes [8].

When discussing dietary fibers, it is essential to consider the involvement of microbiota-derived metabolites able to crosstalk with hormones from the host [9]. Despite the exponential growth in published research, a comprehensive understanding of how to modulate the gut microbiota for the treatment of metabolic and endocrine diseases remains elusive. Future research efforts should prioritize high-quality intervention studies aimed at elucidating these complex interactions.

In this Special Issue, Lim et al. reported the findings of the INTESTINE Study [10], in which they elegantly demonstrated that the gut microbiota influences the efficacy of antidiabetic medications in Korean drug-naïve patients with type 2 diabetes. A combined treatment with gemigliptin–metformin proved more effective in normalizing glycemia and body weight compared to the glimepiride–metformin combination. These improvements were associated with a reduction in the Firmicutes/Bacteroidetes ratio observed in the gemigliptin–metformin group. Indeed, targeting the microbiota and its derived metabolites represents a promising therapeutic strategy to meet the pressing need for novel and effective treatments for type 2 diabetes. Recent preclinical findings have demonstrated that treatment with *Faecalibacterium prausnitzii* significantly ameliorates fasting blood glucose levels, IR, inflammation, and lipid metabolism in type 2 diabetic mice [11].

Fliag et al. [12] conducted a comprehensive review of the evidence regarding the effectiveness of dietary interventions and targeted microbiota therapies for the treatment of dyslipidemia. The beneficial effects were primarily attributed to strategies that enhance the abundance of short-chain fatty acid (SCFA)-producing bacteria, which play a crucial role in maintaining gut barrier integrity [13]. These promising findings should be further validated using longitudinal studies in order to extend their clinical applicability in humans [14]. 

Finally, the study by Marabotto et al. [15] investigated the prevalence of gastrointestinal symptoms and lactose intolerance (LI) in patients with Hashimoto’s thyroiditis. In fact, the absorption of synthetic levothyroxine (LT4) can be influenced by both dietary factors and digestive impairments [16]. Despite LI being significantly prevalent in the study sample, it did not impact the required LT4 dosage and was not identified as a major cause of LT4 malabsorption. Consequently, the study suggests that routine LI screening may not be necessary for all Hashimoto’s thyroiditis patients presenting with gastrointestinal symptoms. 

Overall, the contributions to this Special Issue provided new data to support the strict relationship between diet and lifestyle behaviors in maintaining hormonal homeostasis and human health. 

However, alongside diet and stress management, physical activity is another key component of an individual’s lifestyle that deserves attention due to its capacity to influence the hormonal and metabolic network. A sedentary lifestyle is a major risk factor for metabolic and endocrine diseases [17]. Knowledge of physical inactivity’s contributions to IR and type 2 diabetes is well established [18]; however, more recent findings support a link between IR and neurodegenerative disorders, such as Alzheimer’s disease [19]. Unfortunately, our understanding of the mechanisms underlying these associations remains limited due to the relatively small number of clinical studies.

Additional insights are required in the field of endocrine disruptors (EDs), particularly environmental obesogens, which have the potential to interfere with the normal endocrine regulation of metabolism [20]. EDs may be present in food either as natural constituents (e.g., phytoestrogens) or as contaminants (e.g., pesticides or packaging materials). Variations in environmental exposure can influence metabolic homeostasis and hormonal profiles, as recently demonstrated for Polycystic Ovary Syndrome (PCOS) [21], underscoring the necessity of environmental assessment across diverse settings.

In summary, it is becoming increasingly evident that metabolic and endocrine disorders emerge from the intricate interactions between environmental factors, the microbiota, and the body’s regulatory systems. In the era of biological big data, the need for effective strategies to integrate and interpret this vast amount of information is more pressing than ever. Therefore, we propose framing research rationales, data analysis, and interpretation within the paradigm of psychoneuroendocrineimmunology (PNEI), which emphasizes the bidirectional interactions between the environment and the neuroendocrine and immune systems [22]. This approach holds potential for advancing new diagnostic and therapeutic strategies, ultimately improving clinical outcomes.

## Data Availability

Not applicable.
